# A New Role for *LOC101928437* in Non-Syndromic Intellectual Disability: Findings from a Family-Based Association Test

**DOI:** 10.1371/journal.pone.0135669

**Published:** 2015-08-19

**Authors:** Shaohe Zhou, Zhangyan Shi, Meng Cui, Junlin Li, Zhe Ma, Yuanyu Shi, Zijian Zheng, Fuchang Zhang, Tianbo Jin, Tingting Geng, Chao Chen, Yale Guo, Jianping Zhou, Shaoping Huang, Xingli Guo, Lin Gao, Pingyuan Gong, Xiaocai Gao, Kejin Zhang

**Affiliations:** 1 Key Laboratory of Resource Biology and Biotechnology in Western China (Ministry of Education), College of Life Science, Institute of Population and Health, Northwest University, Xi’an, China; 2 Xi’an Institute of Mental Health, Xi’an, China; 3 College of Public Management, Institute of Application Psychology, Northwest University, Xi’an, China; 4 School of Life Sciences, Northwest University, Xi’an, Shaanxi, China; 5 National Engineering Research Center for Miniaturized Detection Systems, Xi’an, Shaanxi, China; 6 The 2^nd^ Affiliated Hospital, Xi’an Jiaotong University, Xi’an, China; 7 School of Computer Science and Technology, Xidian University, Xi'an Shaanxi, China; 8 Laboratory of Medical Molecular Biology, Henan University of Science and Technology, Luoyang, China; Sudbury Regional Hospital, CANADA

## Abstract

Non-syndromic intellectual disability (NSID) is mental retardation in persons of normal physical appearance who have no recognisable features apart from obvious deficits in intellectual functioning and adaptive ability; however, its genetic etiology of most patients has remained unknown. The main purpose of this study was to fine map and identify specific causal gene(s) by genotyping a NSID family cohort using a panel of markers encompassing a target region reported in a previous work. A total of 139 families including probands, parents and relatives were included in the household survey, clinical examinations and intelligence tests, recruited from the Qinba mountain region of Shannxi province, western China. A collection of 34 tagged single nucleotide polymorphisms (tSNPs) spanning five microsatellite marker (STR) loci were genotyped using an iPLEX Gold assay. The association between tSNPs and patients was analyzed by family-based association testing (FBAT) and haplotype analysis (HBAT). Four markers (rs5974392, rs12164331, rs5929554 and rs3116911) in a block that showed strong linkage disequilibrium within the first three introns of the *LOC* *101928437* locus were found to be significantly associated with NSID (all *P*<0.01) by the FBAT method for a single marker in additive, dominant and recessive models. The results of haplotype tests of this block also revealed a significant association with NSID (all *P*<0.05) using 2-window and larger HBAT analyses. These results suggest that *LOC* *101928437* is a novel candidate gene for NSID in Han Chinese individuals of the Qinba region of China. Although the biological function of the gene has not been well studied, knowledge about this gene will provide insights that will increase our understanding of NSID development.

## Introduction

Intellectual disability (ID), also known as mental retardation, has a 2% prevalence in the population [1]. ID is a common disorder involving significantly lower intellectual function and lower social adaptive ability prior to the age of 18 years. Approximately 30% of individuals with mild ID do not have other obvious clinical syndromes; thus, they are defined as having non-syndromic intellectual disability (NSID) [2]. Approximately 25% of all cases of ID are caused by a genetic disorder, although typically, the etiology of this disability is poorly understood.

Strong evidence from numerous carefully performed studies has suggested that genetic factor is an important causative etiology of NSID which are present in 25–50% of ID patients, and this number increases proportionally with severity [3]. More than 40 specific genes associated with NSID have been identified, and many of these genes are localized on the X chromosome, which may be partly due to the more easy identification of NSID genes in X-linked pedigrees and the high male-to-female ratio (approximately 1.3 to 1.4) [4]. Most of the reported NSID genes are involved in common pathways and processes including the ionotropic glutamate receptors pathway, excitatory synapses function, cell adhesion, the Rho pathway, synaptic vesicle trafficking and exocytosis, zinc finger proteins function, the ERK/MAPK pathway, transcriptional regulation and chromatin remodeling [2]. Certainly, as more NSID genes are identified, more of the common biological pathways that are involved in NSID will be uncovered, which will help us to understand, prevent and alleviate this disorder.

Substantial research has shown that genes on chromosome Xq21-23 may be involved in NSID, and more than five genes in this region are currently known to be mutated in NSID patients and have been reported in more than one unrelated family or X-linked NSID family [4]. However, there are limited reports describing candidate genes that have a strong association with NSID in the Han Chinese population. In addition, we previously reported a strong association between five short tandem repeats (STRs) (DXS7132, DXS1191, DXS1230, DXS1072 and DXS6804) and NSID using a random case-control sample in an isolated Han Chinese population [5]. However, additional fine mapping was needed because more than 100 known genes are included in the regions close to the STRs. We hypothesized that in addition to the previously reported NSID genes, new candidate genes and mutations may be found in this region in individuals from the Qinba region of China. Therefore, replicate studies and refinement work are warranted, especially for genes that have been reported in single/several typical pedigrees or specific ethnic populations [6] and particularly when the need for intervention protocols and methods for the diagnosis and prevention of this disease are considered.

NSID genes have typically been identified in only one individual or family when combining homozygosity mapping with sequencing methods [2]. Family-based population research is clearly superior for elucidating the genetic basis of NSID [7]. In general, association test results can be biased if the sample contains individuals with different ancestries[8], although these tests are commonly used to confirm or exclude relationships between a disease and selected genes. Additionally, population stratification, geographic isolation and other potential elements such as the demographic structure of the population may also exacerbate this problem [9]. The inconsistent and non-repeatable results obtained for most candidate NSID genes using different independent samples also reflect this potential bias [6]. Therefore, based on the results of case-control analyses, fine mapping and/or verification studies with a family cohort by family-based association testing (FBAT) analysis may be a favorable and cost-effective strategy that avoids the potential confounding effects mentioned above [10].

To our knowledge, few NSID candidate gene studies have been conducted with a two-step method using two independent samples with the objective of identifying genetic loci for NSID. In the present study, 139 extended NSID pedigrees of Han Chinese individuals in the Qinba region are included for refinement mapping and replication of associations with NSID.

## Materials and Methods

### Participants and phenotypic assessment

Based on the Chinese Classification of Mental Disorders 2^nd^ Revision (CCMD-2-R), the Diagnostic and Statistical Manual of Mental Disorders (DSM-IV) and the classification used by mental and behavioral clinical psychiatric pediatricians, we diagnosed, identified, and classified ID patients from among more than 20,000 children aged 4 to 16 years. NSID children, identified as probands, as well as their parents and relatives were all tracked, investigated and diagnosed. The intelligence of each child was screened using the Chinese Wechsler Young Children Scale of Intelligence [11] for 4–5-year-olds and the Chinese Wechsler Intelligence Scale for Children [12] for 6–14-year-olds. Social disability (SD) scores were assessed using the Adaptive Scale for Infants and Children revised by Zuo Q [13]. Subsequently, a clinical examination was carried out by a group of neurologists, pediatricians, and gynecologists to evaluate the children with an IQ <85 and SD scores of 9 or lower. Parents and adult family members were tested using the Third Revision of Combined Raven’s Test (CRT-RC3) revised by Qian M [14], the abbreviated version of the Wechsler Adult Intelligence Scale-Chinese Revision (WAIS-RC) [15] and the Adult Mental Handicap Scale (AMHS) [16]. The definition of NSID and the diagnostic criteria were based on the WHO classification of mental and behavioral disorders [17]. Cases of NSID affected by trachoma, infection, trauma, toxicity, cerebral palsy, birth complications, or cretinism/sub-cretinism were excluded. All subjects were identified and recruited from the Qinba mountain region of Shannxi province, western China.

In total, 1499 individuals (349 NSID cases and their relatives), including 748 females and 751 males, with ages ranging from 4 to older than 73 and forming 139 extended pedigrees were tracked, recruited, and investigated. Whole genomic DNA samples were extracted from 1 mL of peripheral blood leukocytes using the genomic DNA extraction kits (for blood) following the manufacturer’s instructions. The DNA concentration was determined by cuvette status measurement and diluted with DNA buffer for genotyping.

All subjects were of Han Chinese descent. The protocol was performed in accordance with the Declaration of Helsinki and approved by the Ethics Committee of Northwest University. Written informed consent was obtained from all participants and/or their guardian.

### SNP selection and genotyping

Single nucleotide polymorphisms (SNPs) were selected to verify the association between NSID and the following five reported microsatellite markers (STRs): DSX7132, DSX1191, DSX1230, DSX1072 and DSX6804. To cover the candidate regions and minimize the genotyping load while maximizing association information, the method of Gabriel et al. [18] was implemented in HaploView 3.2 software [19] with genotype data from HapMap CEU and HBC populations. The candidate regions were defined as 500 kb regions surrounding each STR locus. For this study, 34 tagged SNPs (tSNPs) were included and examined (S1 Table). SNP genotyping was performed using a Sequenom MassARRAY iPLEX Gold assay according to the manufacturer’s instructions. Data management and analysis was performed using Sequenom Typer 4.0 software [20]. SNPs with a successful genotyping rate (>95%) and MAF >0.1 were selected and analyzed.

### Data analysis

Microsoft Visual FoxPro 9.0 software was used to analyze raw data. SNP data from the family samples were tested for Mendelian inconsistencies using the program PEDCHECK [21] prior to re-genotyping and Mendel’ law test analysis. Sub-family pedigrees did not provide statistical information that was useful in association analyses when Mendelian inconsistencies were found, although use of the FBAT approach may increase model misspecification. Deviation from Hardy-Weinberg equilibrium (HWE), the presence of linkage disequilibrium (LD) and blocks consisting of adjacent SNPs showing strong LD among each other were tested using HaploView 3.2 software. Due to the family-based cohort and recruitment from a relatively isolated mountain region, HWE ≤0.001 was considered significant. We tested for excessive transmission of alleles separately at each marker, and we subsequently tested for excessive transmission of multiple-marker haplotypes in each block. Haplotypes were reconstructed using the HBAT command in FBAT for analyses that utilized the EM algorithm. In these analyses, we used the empirical variance estimator option (*-e*) because our primary hypothesis was association in the presence of linkage. Statistical significance was set at *P*<0.05. To reduce the risk of false discoveries, we calculated a q-value for each *P*-value, which is an estimate of the proportion of false discoveries among all significant markers, using QVALUE [22]. The corresponding *P*-value was used as the threshold for declaring significance.

## Results

### Genotyping Profile

In the present study, 34 tSNPs with no significant deviation from HWE distribution were found; however, three SNPs were identified that showed significant deviation: rs6624142 (*P* = 0.001), rs508804 (*P*<0.001) and rs2369623 (*P* = 0.001) (S2 Table). Two sites, rs7889957 and rs10465337, showed low heterozygosity (Het. <0.15) and fewer informative families, although they showed good heterozygosity based on genotype data from HapMap CEU and HBC populations. In the subsequent FBAT analysis of the SNPs, significant deviation from HWE and poor heterozygosity will be considered.

### LD Structure of the Target Region

To determine the LD relationship among SNPs in and around the five STR loci, LD structure analysis was performed using HaploView software. Three blocks with high LD distribution were found in our samples, which was in line with the result of LD distribution analysis with HapMap data set. Four SNPs near DXS1191, (rs5945714, rs5945866, rs4826940 and rs1044311) were located in block 1, and the other three SNPs (rs6622044, rs2754830 and rs6622104) were in block 2. Five SNPs (rs3125999, rs3116911, rs5929554, rs12164331 and rs5974392) near DXS1072 and DXS6804 also showed a stronger LD relationship in block 3 (chrX: 112,140–112,309 kb, from 5kb upstream (5’) of rs3125999 to 5 kb downstream (3’) of rs5974392) (S1 Fig). Haplotype analysis was performed within these three blocks.

### Association Analysis of Markers with NSID

Single marker association analysis revealed that seven SNPs were significantly associated with NSID (Table 1) in our family cohort in the dominant model, both with general and–*e* option analyses of the FBAT method. Furthermore, the same results were obtained in the additive and recessive models (data not shown). The T allele of rs6624142 displayed a negative association (Z = -2, *P*_*e*_ = 0.046) with NSID, whereas the A allele of rs6622044 and the G allele of rs4829463 showed significant positive associations (Z = 2.24, *P*_*e*_ = 0.025; Z = 2.6, *P*_*e*_ = 0.09, respectively). Four other SNPs located in block 3, rs3116911, rs5929554, rs12164331 and rs5974392, also showed a strong association with NSID (Z = 2.89, *P*_*e*_ = 0.004; Z = 3.27, *P*_*e*_ = 0.001; Z = 3.67, *P*_*e*_<0.001; and Z = 3.13, *P*_*e*_ = 0.002, respectively). One of the alleles from these SNPs was over-transmitted in families with one or more NSID patients. Only rs6624142 and rs6622044 did not remain significant after multiple corrections using FDR and 1000 permutation test runs of the FBAT analysis.

**Table 1:** Family-based associate test for tSNPs with dominant model.

Marker ^a^	Alleles ^b^	Allele Frequency	Test Statistic (Z)	*P*-FBAT	*P*_*e*_-FBAT
rs6624142	T/C	.84/.16	-2	**.032** ^**c**^	**.046** ^**c**^
rs221946	G/A	.46/.54	0.78	.414	.433
rs6523754	T/C	.37/.63	0.73	.480	.465
rs508804	C/T	.63/.37	-1.39	.166	.166
rs5945714	A/G	.67/.33	0.39	.705	.695
rs5945866	A/G	.51/.49	1.73	.083	.083
rs4826940	G/T	.32/.68	-0.38	.724	.705
rs1044311	T/C	.72/.28	-0.6	.564	.548
rs1323219	T/G	.18/.82	-0.23	.835	.818
rs1323223	T/C	.29/71	0.85	.433	.394
rs169677	A/C	.27/73	1.28	.273	.201
rs7056233	A/G	/.14	-1.16	.317	.248
rs5916965	C/T	.75/.25	-0.45	.695	.655
rs5962312	T/C	.81/.19	-0.43	.683	.670
rs6622044	A/C	.62/.38	2.24	**.041** ^**c**^	**.025** ^**c**^
rs2754830	A/C	.84/.16	-1.60	.180	.109
rs6622104	G/A	.54/.46	-0.82	.273	.414
rs1426860	A/T	.30/.70	0.2	.847	.841
rs1991340	C/T	.44/.56	1	.411	.317
rs2880013	C/A	.43/.57	0	1	1
rs11152711	G/A	.39/.61	1.98	.070	.048
rs583430	A/T	.21/.79	-0.58	.532	.564
rs650005	T/C	.80/.20	-0.38	.695	.705
rs478739	G/C	.60/.40	-0.49	.602	.622
rs4829463	G/A	.63/.37	2.6	**.004** ^**c**^	**.009** ^**c**^
rs6568109	T/G	.82/.18	-1.07	.285	.285
rs3125999	C/A	.85/.15	-0.28	.782	.782
rs3116911	G/A	.54/.46	2.89	**.002** ^**c**^	**.004** ^**c**^
rs5929554	T/A	.67/.33	3.27	**.0004** ^**c**^	**.001** ^**c**^
rs12164331	C/T	.56/.44	3.67	**.0001** ^**c**^	**.0002** ^**c**^
rs5974392	T/G	.77/.23	3.13	**.001** ^**c**^	**.002** ^**c**^
rs2369623	C/T	.64/.36	-0.69	.513	.491

Abbreviations: FBAT, Family Based Association Test; *P*-FBAT, p values of FBAT test; *P*_*e*_-FBAT, significance test by FBAT with –*e* option.

^a^ Two SNPs, SNP2 and SNP3, did not include because of poor informative families for their poor heterozygosities (>0.15).

^b^ For SNPs, alleles shown are those for which there were more than 10 informative families.

^c^ Significant P values (<0.05) are bold.

### Haplotype Analyses in Blocks

**Fig 1:**
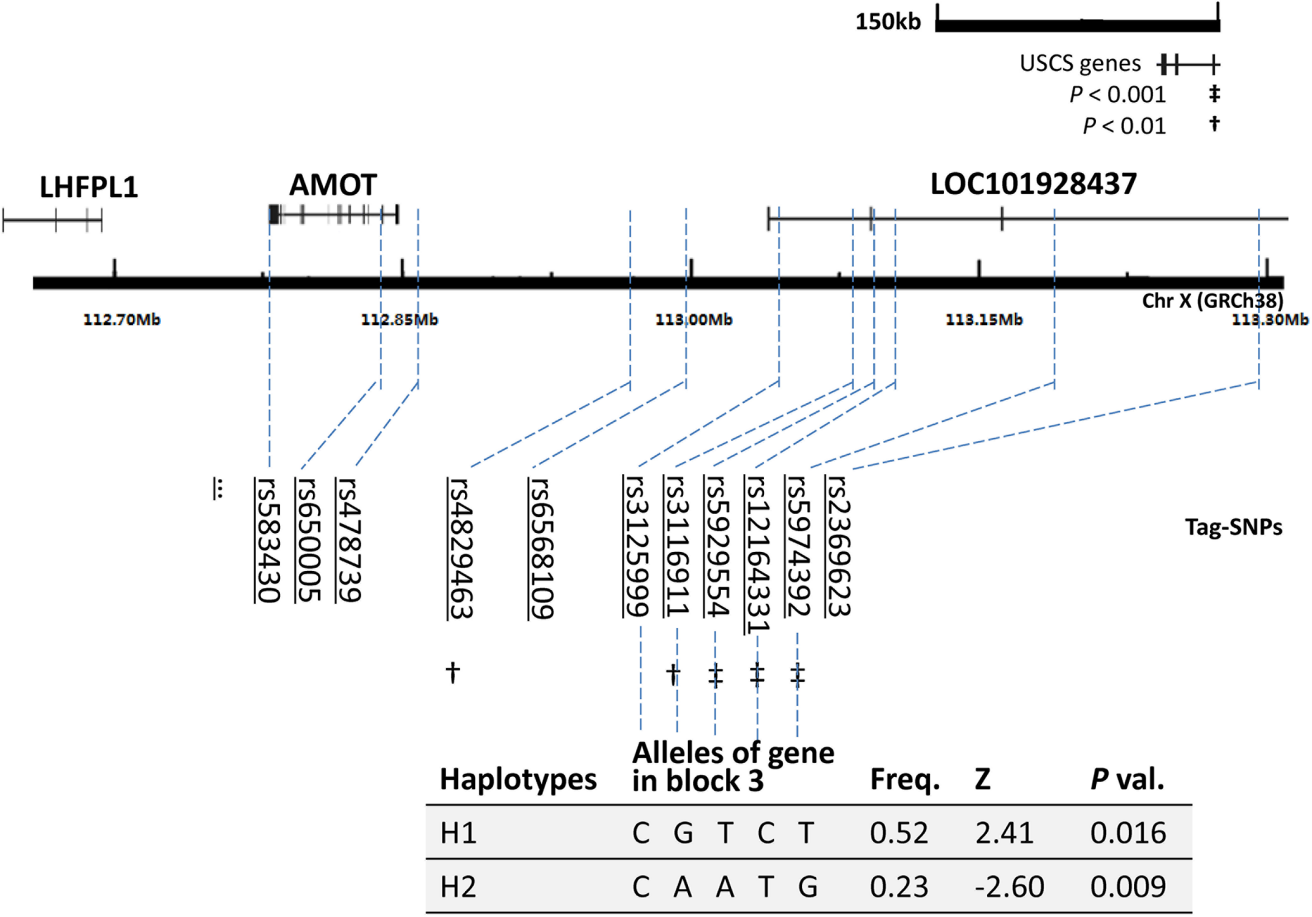
Markers near BLOCK3 that were included in association analysis. The names and relative positions of the markers are shown along with their relationships with the *LOC101928437* gene and the adjacent genes *AMOT* and *LHFPL1*. Five marker haplotypes with above 5% frequency are shown. *LHFPL1*, lipoma HMGIC fusion partner-like 1 gene; *AMOT*, angiomotin gene; *LOC101928437*, *LOC101928437* gene.

Haplotype analyses were performed using HBAT methodology with 2- and 3-window sizing for blocks 1, 2, and 3. A significant association was only found in block 3. Only the 2-window haplotype structure analysis showed the dominant alleles (frequency >0.15) for each haplotype, which included one or two positively associated SNPs, as listed in Table 2. However, some of the haplotypes consisting of unfavorable alleles still showed significant associations with NSID (all *P*_*e*_ <0.05) (S3 Table). Similar results were recorded with the 3-, 4- and 5-window methods (all *P*_*e*_ <0.05). For all five marker haplotype analyses, there was one site (the C allele of rs3125999) that remained the same for both haplotype H1 and H2 and that demonstrated positive/negative association (H1, Z = 2.41, *P* = 0.016; H2, Z = -2.60, *P* = 0.009, respectively) with NSID (Fig 1). Haplotypes with dominant frequencies (>15%) exhibited significant over-transmission for every SNP combination. The global *P* values were also significant. During window analyses of the same size, halpotypes containing rs3125999 demonstrated a weak association with NSID compared to others without this marker. All positive results were multiple-test corrected with FDR and 1000 permutation test runs.

**Table 2:** Haplotype (2-window sized) analysis constructed with positive tSNPs within BLOCK 3.

Haplotypes	Frequency ^a^	Var (S)	Test Statistics (Z)	*P*-HBAT ^b^	*P*_*e*_-HBAT ^b^	Global *P* ^b^ ^,^ ^c^
rs3125999/ rs3116911						**.016**
H1	.533	7.75	1.98	**.048**	**.048**	
H2	.311	9.25	-2.14	**.024**	**.032**	
rs3116911/ rs5929554						**< .001**
H1	.49	8.25	2.26	**.024**	**.020**	
H2	.30	8.25	-2.61	**.009**	**.014**	
H3	.17	4.00	1.00	.317	.248	
rs5929554/ rs12164331						**< .001**
H1	.50	8.25	2.26	**.024**	**.020**	
H2	.27	7.5	-2.92	**.003**	**.005**	
H3	.17	4	1.00	.317	.248	
rs12164331/ rs5974392						**< .001**
H1	.54	7.75	2.34	**.020**	**.012**	
H2	.22	6.5	-2.75	**.006**	**.010**	
H3	.20	5.2	.22	.827	.800	

Abbreviations: *P*-HBAT, p values of HBAT test; *P*_*e*_-HBAT, significance HBAT test with –*e* option; Global *P*, p values of the asymptotic global HBAT test for all haplotypes with more than 0.05 frequencies and within one window or block.

^a^ For haplotypes with higher frequencies (>15%) and more than 10 informative families were shown;

^b^ Significant *P* values (<0.05) are bold;

^c^ haplotypes with frequencies > 0.05 were included for global *P* test.

## Discussion

In the present study, we sought to clarify the associations among five STR markers located on the X chromosome and conduct fine mapping using independent family cohort samples. After testing 34 tSNPs to determine a potential association with NSID, we identified 5 tSNPs that showed significant over-transmission among NSID patients and their relatives. Four of these tSNPs (rs3116911, rs5929554, rs12164331 and rs5974392) are located adjacent to each other and are significantly associated with NSID in both single-site and haplotype analyses among Han Chinese from the Qinba region of China. Haplotype structure analysis suggested that all the positive SNPs are located within/near BLOCK3 (chrX: 112,140..112,309 kb) and cover a 200 kb sequence (Fig 1). There are no reports of NSID genes located in or near this region. In this NSID extended family cohort, we observed that some causative NSID genetic elements may be located in or near this region (BLOCK3). However, it is likely that more than one gene is involved, thus further studies are needed.

*LOC101928437* (Gene ID: 101928437), a non-coding RNA (ncRNA) gene, may be a new NSID gene in Han Chinese individuals from the Qinba region because it shows an overlap with BLOCK3 (Fig 1). The four SNPs associated with NSID, rs3116911, rs5929554, rs12164331 and rs5974392, are located in the first three introns of *LOC101928437*. A bi-colored network-based global function prediction analysis also indicated that the *LOC101928437* gene overlaps with a human large intergenic non-coding RNA (lincRNA) gene which shows a high level of specific expression in the brain (S4 Table). In theory, this lincRNA gene may be involved in the etiology of NSID via coexpression with neighboring genes at a level similar to that of the neighboring protein-coding genes [23]. Therefore, we cannot rule out a potential role for this lincRNA in the etiology of NSID, although its characterization and biological function are limited thus far. Furthermore, *in silico* analysis results suggested that the alternative alleles of the four SNPs have certain potential biological functions (S1 File and S5 Table), although all of these SNPs are located in introns. The influences of these SNPs on the function of *LOC101928437* are still unknown, and direct evidence of the association of *LOC101928437* gene variants with NSID has not yet been obtained; thus, greater knowledge about these factors will help to elucidate the association between this gene and NSID.

As substantial research suggested that, X chromosome still has an enrichment of mental retardation syndrome and other psychiatric disorders [24, 25]. Three genes, including *AMOT* (Gene ID: 154796, chrX: 112,018..112,084 kb), *LHFPL1* (Gene ID: 340596, chrX: 111,873..111,923 kb) and *ZCCHC16* (Gene ID:340595, chrX:111,326..111,700 kb), are nearby BLOCK 3. It seemed not the candidate NSID gene in current cohort, although certain indirect evidences showed their relationship with human psychiatric disorders [5, 26–29]. Firstly, both Fig 1 and S1 Fig LD structures indicated that, all of these gene are located in other independent block rather than BLOCK 3. Furthermore, an additional LD structure analysis with another independent sample data collected from HapMap with 90 individuals also showed the same result (data not shown). Secondly, four SNPs which had significant association with NSID both in single-site and haplotype analysis were all located in the first three intronx of *LOC101928437* gene, and in BLOCK 3 simultaneously. Although, rs6624142 and rs6622044 also showed a weak association with NSID in single marker analysis (Table 1), the results were not sustained after Bonferroni correction (http://www.quantitativeskills.com), and the subsequent multimarker haplotype analysis also failed to identify this trend.

In the current study, we report an association between an X chromosome region (Xq23) and NSID in a Han Chinese family-based cohort from the Qinba region of China. The current literature has identified several NSID candidate genes located in or near this region (Xq21-23) [6]; however, with the exception of the *LOC101928437* gene, no genes close to BLOCK3 have been reported to show a consistent association using either family-based cohort or random case-control population samples. Associations between the genes examined in this paper and NSID have not been reported previously. Further work should be performed to obtain direct evidence linking this gene with NSID and to understand the influence of coexpression and interaction of this gene with neighboring genes on NSID, especially considering the features of lincRNAs.

This study involved a two-step association analysis with different independent populations. First, we identified candidate genes using random case-control samples to detect potentially associated loci. Then, we performed fine mapping within a family-based cohort population sample to clarify the association of these candidate genes with NSID. Consistency between the single marker and multimarker haplotype analyses was assessed to clarify the results. The FBAT method incorporates almost all subjects who are investigated, phenotyped and genotyped, and although parental genotype information was not obtained in this study, this method is more powerful, robust and effective compared with the typical familiar transmission/disequilibrium test (TDT) and linkage analysis of single or multiple families [30, 31]. Additionally, the *–e* option of FBAT can provide an empirical correction for the variance of the statistical test [32]. *In silico* analyses of the sequences surrounding each SNP (especially for SNPs located in introns or non-coding sequences with unknown functions) were used to predict the potential function of the markers for identifying candidate genes. In the present study, we identified relationships between genetic elements and NSID that were highly statistically significant with a high level of confidence.

Our study also has limitations. First, although the association between BLOCK 3, which harbors *LOC101928437*, and NSID was supported by highly consistent FBAT single-site and haplotype analyses, more robust evidence (e.g., mutation scanning, sequencing or related bio-functional analyses) is needed. Second, any potential relationships between genes that are located close to BLOCK 3 and NSID should be ruled out, as we cannot rely solely on the LD structure analysis results. For example, targeted sequencing that focuses on BLOCK3 and nearby genes may be an applicable strategy in future work. Finally, this was an isolated population study; therefore, the results and conclusion of our study should be validated in other independent populations.

In summary, we have identified a significant association between *LOC101928437* and NSID in the Qinba region of China. Further studies are needed to clarify the function of the locus and to determine how variations in this locus influence the development of NSID.

## Supporting Information

S1 Fig LD structure of SNPs in a family-based cohort. LD blocks were identified using the 4-gamete rule as implemented in Haploview. The magnitude of LD indexed by the *D’* statistic is also shown. Red squares without numbers indicate complete LD (*D’* = 1). *D’* values are given in the squares for values <1.0. Adjacent SNPs showing higher LD (*D’* ≥0.8) were defined as one black and marked with a black frame.
(TIF)

S1 File Supporting information. (DOCX)

S1 Table Location and allele information for the tagged SNPs examined in this study. (DOCX)

S2 Table The profile of tagged SNPs and the results of the Hardy-Weinberg disequilibrium test. (DOCX)

S3 Table Haplotype analysis with positive SNPs within BLOCK 3. (DOCX)

S4 Table Bi-colored network based global function prediction and tissue-specific expression analysis for the *LOC1019288437* sequence. (DOCX)

S5 Table *In silico* analysis results for SNPs with significant associations in single marker analysis. (DOCX)
