# Supplementary figures and images for: A New Role for LOC101928437 in Non-Syndromic Intellectual Disability: Findings from a Family-Based Association Test
Source: PLoS One. 2015 Aug 19;10(8):e0135669. doi: 10.1371/journal.pone.0135669 (PMC4545728; doi:10.1371/journal.pone.0135669)

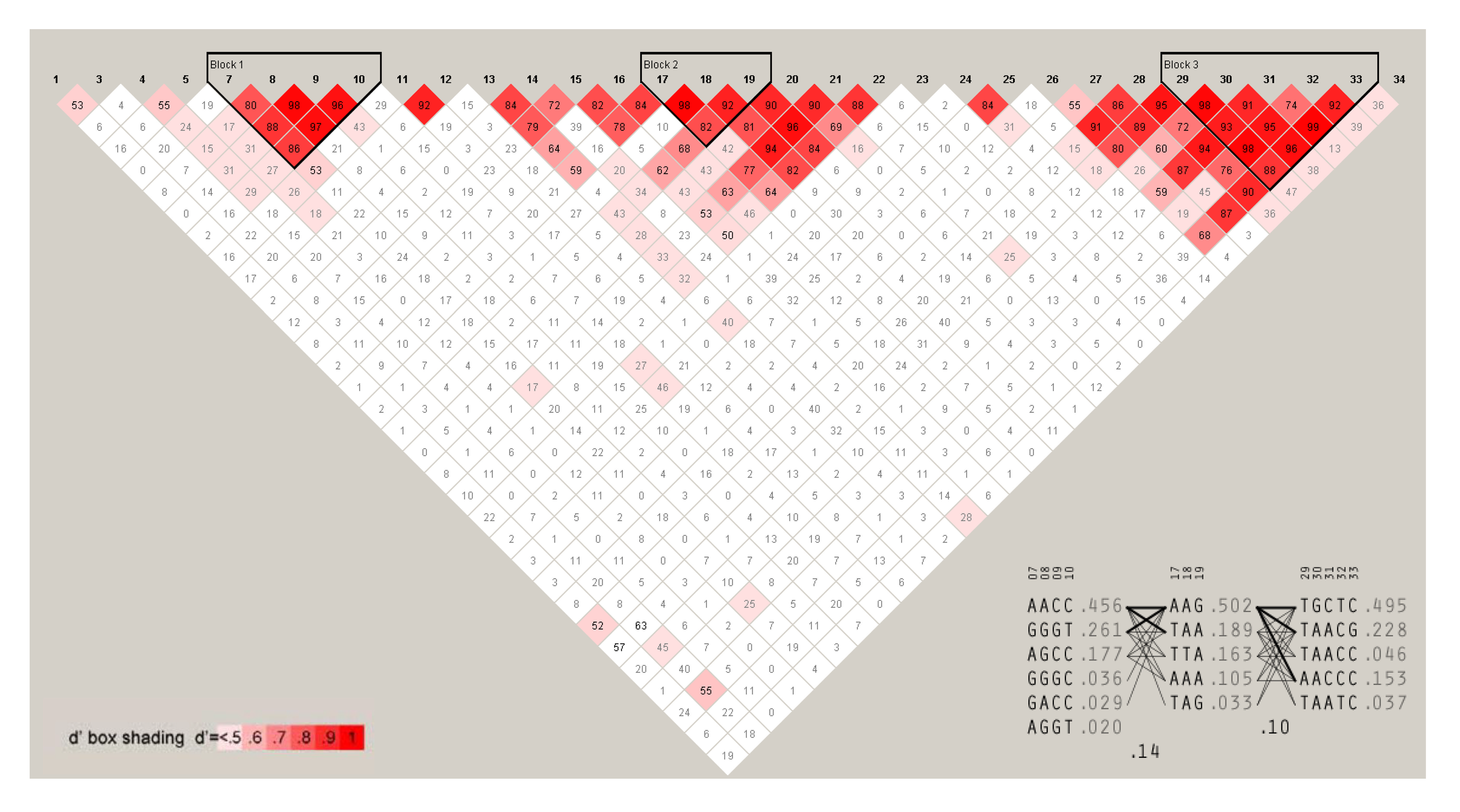

Supplement: S1 Fig — LD blocks were identified using the 4-gamete rule as implemented in Haploview. The magnitude of LD indexed by the D’ statistic is also shown. Red squares without numbers indicate complete LD (D’ = 1). D’ values are given in the squares for values <1.0. Adjacent SNPs showing higher LD (D’ ≥0.8) were defined as one black and marked with a black frame. (TIF) [file pone.0135669.s001.tif]
