# Supplementary material for: A New Role for LOC101928437 in Non-Syndromic Intellectual Disability: Findings from a Family-Based Association Test
Source: PLoS One. 2015 Aug 19;10(8):e0135669. doi: 10.1371/journal.pone.0135669 (PMC4545728; doi:10.1371/journal.pone.0135669)
Supplement: S1 File — (DOCX) [file pone.0135669.s002.docx]

**Supporting Information**

**A new role for *LOC101928437* in non-syndromic intellectual disability: findings from a family-based association test**

Shaohe Zhou ^1^, Zhangyan Shi ^1^, Meng Cui ^2^, Junlin Li ^1^, Zhe Ma ^1^, Yuanyu Shi ^1^, Zijian Zheng ^3^, Fuchang Zhang ^1, 3^, Tianbo Jin ^4, 5^, Tingting Geng ^4, 5^, Chao Chen ^4, 5^, Yale Guo ^6^, Jianping Zhou ^6^, Shaoping Huang ^6^, Xingli Guo ^7^, Lin Gao ^7^, Pingyuan Gong ^8^, Xiaocai Gao ^1, 3 *^, Kejin Zhang ^1 *^

^1^ Key Laboratory of Resource Biology and Biotechnology in Western China (Ministry of Education), College of Life Science, Institute of Population and Health, Northwest University, Xi’an, China; ^2^ Xi’an Institute of Mental Health, Xi’an, China; ^3^ College of Public Management, Institute of Application Psychology, Northwest University, Xi’an, China; ^4^ School of Life Sciences, Northwest University, Xi’an, Shaanxi, China; ^5^ National Engineering Research Center for Miniaturized Detection Systems, Xi’an, Shaanxi, China; ^6^ The 2^nd^ Affiliated Hospital, Xi’an Jiaotong University, Xi’an, China; ^7^ School of Computer Science and Technology, Xidian University, Xi'an Shaanxi , China; ^8^ Laboratory of Medical Molecular Biology, Henan University of Science and Technology, Luoyang, China

**Supplementary materials and methods**

***In silico* analysis for positive SNPs**

VISTA was used to define the conserved regions in the genomic sequence covered by haplotypes associated with NSID with statistically significant *P*-value, and visualized as added tracks on the University of California Santa Cruz genome browser (<http://genome.ucsc.edu/>). *In* *Silico* analyses were performed to estimate the potential biological function of SNPs based on three aspects: 1) the transcription factor binding site or a promoter site analysis; 2) the conservative property estimation and the splice site; and 3) a new protein structure or function resulted directly from the target SNPs. Briefly, TFSEARCH program (<http://www.cbrc.jp/research/db/TFSEARCH.html>) was used to search the transcription factor-binding (TFB) sites with vertebrate classification and a threshold score of 85.0 [[1](#_ENREF_1)], within 50bp sequence surrounding each SNP. The promoter predictions of Neural Network Promoter Prediction, Promoter 2.0 Prediction and McPromoter MM:II were performed to identify whether there were certain promoter sites generated or loss because of alternative SNP alleles. Splice site estimation also performed with MasEntScan software based on the maximum entropy principle [[2](#_ENREF_2)], and Splice Site Predictor from the Berkeley Drosophila Genome Project with Neural Network method [[3](#_ENREF_3)]. Additional 300bp sequences surrounding the target SNPs were analyzed by ORF-finder (<http://www.ncbi.nlm.nih.gov/gorf/gorf.html>) and CCD (<http://www.ncbi.nlm.nih.gov/Strucure/cdd/wrpsb.cgi>) to identify the possible open reading frames, the amine acid sequence and similar protein families. Phobius (<http://www.ebi.ac.uk/Tools/pfa/phobius/>) and InterProScan (<http://www.ebi.ac.uk/interpro/>) tools evaluated the effect of SNP site alternative on the polypeptide chain’s structure.

**Supplementary result**

**Result of *in silico* analysis for four positive SNPs**

The ORF-finder and CDD analysis indicated that, sequence surrounding rs3116911 may encode a 40aa chain that harbors a transmemberane and TMhelix conservative domain, and it may be a nonsynonymous SNP (Ile>Val). MasEntScan software based on the maximum entropy principle [[2](#_ENREF_2)] and Splice Site Predictor from the Berkeley Drosophila Genome Project with Neural Network method [[3](#_ENREF_3)] also indicated that, the allelic changes of rs5929554 and rs5974392 will influence an acceptor splice sites. Also, rs5929554 may be a nonsynonymous SNP (Glu>Val) within a 64aa polypeptide chain with an higher similarity feature for the transposase_22 multi-domains (L1 transposable element) (S5 Table).

1. Heinemeyer T, Wingender E, Reuter I, Hermjakob H, Kel AE, Kel OV, et al. Databases on Transcriptional Regulation: TRANSFAC, TRRD, and COMPEL. Nucleic Acids Res. 1998;26:7.

2. Yeo G, Burge CB. Maximum entropy modeling of short sequence motifs with applications to RNA splicing signals. Journal of Computational Biology. 2004;11(2-3):377-94.

3. Reese MG, Eeckman FH, Kulp D, Haussler D. Improved splice site detection in Genie. Journal of Computational Biology. 1997;4(3):311-23.
