# Supplementary material for: A New Role for LOC101928437 in Non-Syndromic Intellectual Disability: Findings from a Family-Based Association Test
Source: PLoS One. 2015 Aug 19;10(8):e0135669. doi: 10.1371/journal.pone.0135669 (PMC4545728; doi:10.1371/journal.pone.0135669)
Supplement: S1 Table — (DOCX) [file pone.0135669.s003.docx]

**S1 Table. Location and allele information for the tagged SNPs examined in this study.**

| **SNP ^a^** | **Alleles** | **Physical_pos ^b^** | | **Genetic_pos ^c^** | | **STR ^d^** | |
| --- | --- | --- | --- | --- | --- | --- | --- |
| rs6624142 | C/T | 65185289 | q12 | | DXS7132 | |  |
| rs7889957 | G/T | 65624420 |  |  |  |  |  |
| rs10465337 | A/G | 65803565 |  |  |  |  |  |
| rs221946 | C/T | 103576289 | q22.2 | | DXS1191 | |  |
| rs6523754 | C/T | 103706346 |  |  |  |  |  |
| rs508804 | C/T | 103785171 |  |  |  |  |  |
| rs5945714 | A/G | 103857263 |  |  |  |  |  |
| rs5945866 | A/G | 103955917 |  |  |  |  |  |
| rs4826940 | G/T | 104049400 |  |  |  |  |  |
| rs1044311 | A/G | 104119958 |  |  |  |  |  |
| rs1323219 | G/T | 104435681 |  |  |  |  |  |
| rs1323223 | A/G | 104480322 | q22.2 | | DXS1230 | |  |
| rs169677 | G/T | 105958875 |  |  |  |  |  |
| rs7056233 | A/G | 106023662 |  |  |  |  |  |
| rs5916965 | C/T | 106049399 |  |  |  |  |  |
| rs5962312 | C/T | 106088839 |  |  |  |  |  |
| rs6622044 | A/C | 106120155 |  |  |  |  |  |
| rs2754830 | A/C | 106222614 |  |  |  |  |  |
| rs6622104 | A/G | 106668757 |  |  |  |  |  |
| rs1426860 | A/T | 106671648 |  |  |  |  |  |
| rs1991340 | A/G | 106693176 |  |  |  |  |  |
| rs2880013 | G/T | 106773321 |  |  |  |  |  |
| rs11152711 | A/G | 112474443 | q22.3 | | DXS1072  DXS6804 | |  |
| rs583430 | A/T | 112777464 |  |  |  |  |  |
| rs650005 | C/T | 112831899 |  |  |  |  |  |
| rs478739 | C/G | 112855432 |  |  |  |  |  |
| rs4829463 | A/G | 112970974 |  |  |  |  |  |
| rs6568109 | G/T | 113006596 |  |  |  |  |  |
| rs3125999 | A/C | 113067548 |  |  |  |  |  |
| rs3116911 | A/G | 113075146 |  |  |  |  |  |
| rs5929554 | A/T | 113089796 |  |  |  |  |  |
| rs12164331 | C/T | 113118620 |  |  |  |  |  |
| rs5974392 | G/T | 113174337 |  |  |  |  |  |
| rs2369623 | C/T | 113298546 |  |  |  |  |  |

Abbreviations: a. SNP, the code number of SNP in NCBI dataset; b. Physical_pos: physical position in GRCh38 genomic data package; b. Genetic_pos, genetic position; c. STR, short tandem repeats reported, previously.
