# Supplementary material for: A New Role for LOC101928437 in Non-Syndromic Intellectual Disability: Findings from a Family-Based Association Test
Source: PLoS One. 2015 Aug 19;10(8):e0135669. doi: 10.1371/journal.pone.0135669 (PMC4545728; doi:10.1371/journal.pone.0135669)
Supplement: S2 Table — (DOCX) [file pone.0135669.s004.docx]

**S2 Table.** **The profile of tagged SNPs and the results of the Hardy-Weinberg disequilibrium test.**

| **SNPs** | **ObsHET ^a^** | **HWpval ^b^** | **FamTrio ^c^** | **MendErr ^d^** | **MAF ^e^** | **Alleles** |
| --- | --- | --- | --- | --- | --- | --- |
| rs6624142 | .219 | **.001** | 92 | 0 | .161 | C:T |
| rs7889957 | .0 | 1.0 | 102 | 0 | .0 | G:T |
| rs10465337 | .131 | .920 | 94 | 0 | .079 | G:A |
| rs221946 | .459 | .286 | 79 | 0 | .455 | A:G |
| rs6523754 | .511 | .155 | 89 | 0 | .372 | T:C |
| rs508804 | .766 | **<.001** | 16 | 0 | .368 | T:C |
| rs5945714 | .453 | .968 | 89 | 0 | .33 | A:G |
| rs5945866 | .5 | 1.0 | 78 | 0 | .50 | A:G |
| rs4826940 | .441 | .686 | 92 | 0 | .334 | C:G |
| rs1044311 | .385 | .20 | 90 | 0 | .288 | C:T |
| rs1323219 | .29 | .774 | 92 | 0 | .178 | G:C |
| rs1323223 | .418 | .498 | 91 | 0 | .289 | T:C |
| rs169677 | .42 | .902 | 88 | 0 | .273 | T:A |
| rs7056233 | .257 | .519 | 95 | 0 | .144 | A:G |
| rs5916965 | .409 | .283 | 86 | 0 | .248 | T:C |
| rs5962312 | .339 | .283 | 91 | 0 | .199 | C:T |
| rs6622044 | .464 | 1.0 | 89 | 0 | .385 | A:T |
| rs2754830 | .243 | .284 | 91 | 0 | .168 | A:T |
| rs6622104 | .45 | .162 | 93 | 0 | .452 | G:A |
| rs1426860 | .366 | .029 | 88 | 0 | .298 | C:A |
| rs1991340 | .473 | .507 | 88 | 0 | .442 | C:T |
| rs2880013 | .461 | .373 | 91 | 0 | .426 | A:T |
| rs11152711 | .522 | .185 | 90 | 0 | .395 | A:G |
| rs583430 | .318 | .338 | 92 | 0 | .214 | C:A |
| rs650005 | .334 | .794 | 95 | 0 | .198 | C:T |
| rs478739 | .437 | .121 | 88 | 0 | .399 | G:T |
| rs4829463 | .493 | .697 | 92 | 0 | .377 | G:A |
| rs6568109 | .265 | .558 | 97 | 0 | .179 | C:G |
| rs3125999 | .239 | .813 | 91 | 0 | .152 | T:A |
| rs3116911 | .439 | .050 | 84 | 0 | .478 | G:A |
| rs5929554 | .5 | .172 | 89 | 0 | .341 | C:A |
| rs12164331 | .451 | .143 | 84 | 0 | .451 | T:C |
| rs5974392 | .449 | .004 | 94 | 0 | .243 | C:G |
| rs2369623 | .369 | **.001** | 78 | 0 | .367 | T:C |

Abbreviations: a. ObsHET, SNPs’ observed heterozygosity; b. HWpval, p values of Hardy-Weinberg disequilibrium test, and the cut-off of significant was equal or lower 0.001 in this study; c. FamTrio, informative trio-families count; d. MendErr, Mendelian inconsistence count; e. MAF, minimum allele frequency. The SNPs whose HWpval > 0.001 were included for next step analyses. Significant *P* values and heterozygosity lower than 0.15 are bold.
