# Supplementary material for: A New Role for LOC101928437 in Non-Syndromic Intellectual Disability: Findings from a Family-Based Association Test
Source: PLoS One. 2015 Aug 19;10(8):e0135669. doi: 10.1371/journal.pone.0135669 (PMC4545728; doi:10.1371/journal.pone.0135669)
Supplement: S3 Table — (DOCX) [file pone.0135669.s005.docx]

**S3 Table. Haplotype analysis with positive SNPs within BLOCK 3.**

| **Haplotypes** | **Frequency ^a^** | **Var (S)** | **Test Statistics (Z)** | ***P*-HBAT ^b^** | ***P_-e_* -HBAT ^b^** | **Global**  ***P* ^b, c^** |
| --- | --- | --- | --- | --- | --- | --- |
| rs3125999/ rs3116911 | | | | | | **.016** |
| H1 | .533 | 7.75 | 1.98 | **.048** | **.048** |  |
| H2 | .311 | 9.25 | -2.14 | **.024** | **.032** |  |
| s3116911/ rs5929554 | | | | | | **<.001** |
| H1 | .49 | 8.25 | 2.26 | **.024** | **.020** |  |
| H2 | .30 | 8.25 | -2.61 | **.009** | **.014** |  |
| H1 | .17 | 4.00 | 1.00 | .317 | .248 |  |
| rs5929554/ rs12164331 | | | | | | **<.001** |
| H1 | .50 | 8.25 | 2.26 | **.024** | **.020** |  |
| H2 | .27 | 7.5 | -2.92 | **.003** | **.005** |  |
| H3 | .17 | 4 | 1.00 | .317 | .248 |  |
| rs12164331/ rs5974392 | | | | | | **<.001** |
| H1 | .54 | 7.75 | 2.34 | **.020** | **.012** |  |
| H2 | .22 | 6.5 | -2.75 | **.006** | **.010** |  |
| H3 | .20 | 5.2 | .22 | .827 | .800 |  |
|  |  |  |  |  |  |  |
| rs3125999/ rs3116911/ rs5929554 | | | | | | **.004** |
| H1 | .51 | 7.50 | 2.19 | **.028** | **.028** |  |
| H2 | .28 | 8.00 | -2.48 | **.013** | **.020** |  |
| rs3116911/ rs5929554/ rs12164331 | | | | | | **<.001** |
| H1 | .50 | 8.00 | 2.48 | **.013** | **.011** |  |
| H2 | .26 | 7.25 | -2.78 | **.005** | **.007** |  |
| H3 | .17 | 3.75 | 1.29 | .197 | .132 |  |
| rs5929554/ rs12164331/ rs5974392 | | | | | | **<.001** |
| H1 | .51 | 8.25 | 2.26 | **.023** | **.020** |  |
| H2 | .23 | 6.50 | -2.75 | **.006** | **.011** |  |
| H3 | .16 | 4.00 | 1.00 | .317 | .248 |  |
|  |  |  |  |  |  |  |
| rs3125999/ rs3116911/ rs5929554/ rs12164331 | | | | | | **.001** |
| H1 | .52 | 7.25 | 2.41 | **.016** | **.016** |  |
| H2 | .25 | 7.00 | -2.65 | **.008** | **.011** |  |
| rs3116911/ rs5929554/ rs12164331/ rs5974392 | | | | | | **<.001** |
| H1 | .52 | 8.00 | 2.48 | **.013** | **.011** |  |
| H2 | .23 | 6.50 | -2.75 | **.006** | **.011** |  |
| H3 | .16 | 3.75 | 1.29 | .197 | .132 |  |
|  |  |  |  |  |  |  |
| rs3125999/ rs3116911/ rs5929554/ rs12164331/ rs5974392 | | | | | | **.001** |
| H1 | .52 | 7.25 | 2.41 | **.016** | **.016** |  |
| H2 | .23 | 6.25 | -2.60 | **.009** | **.016** |  |

Abbreviations: *P*-HBAT, p values of HBAT test; *P_-e_* -HBAT, significance HBAT test with –e option; Global *P*, p values of the asymptotic global HBAT test for all haplotypes with more than 0.05 frequencies and within one window or block.

^a^ For haplotypes with higher frequencies (>15%) and more than 10 informative families were shown;

^b^ Significant *P* values (<0.05) are bold;

^c^ haplotypes with frequencies > 0.05 were included for global p test.
