# Supplementary material for: A New Role for LOC101928437 in Non-Syndromic Intellectual Disability: Findings from a Family-Based Association Test
Source: PLoS One. 2015 Aug 19;10(8):e0135669. doi: 10.1371/journal.pone.0135669 (PMC4545728; doi:10.1371/journal.pone.0135669)
Supplement: S4 Table — (DOCX) [file pone.0135669.s006.docx]

**S4 Table. Bi-colored network based global function prediction and tissue-specific expression analysis for the *LOC1019288437* sequence.**

|  | **Specific expression tissues** | | | | | | | | | | | | |
| --- | --- | --- | --- | --- | --- | --- | --- | --- | --- | --- | --- | --- | --- |
| **NONCODE ID** | adipose | adrenal | brain | Breast | colon | foreskin | heart | hela_R | HLF_1 | HLF_2 | kidney | liver | lung |
| **NONHSAT138214** | 0 | 0 | **.007** | 0 | 0 | 0 | **.004** | 0 | 0 | **0.113** | 0 | 0 | 0 |

|  | **Specific expression tissues** | | | | | | | | |
| --- | --- | --- | --- | --- | --- | --- | --- | --- | --- |
| **NONCODE ID** | Lymph Node | ovary | placenta_R | prostate | Skeltal Muscle | testes | testes_R | thyroid | White Blood Cell |
| **NONHSAT138214** | 0 | 0 | 0 | 0 | 0 | 0 | 0 | 0 | 0 |

Abbreviations: NONHSAT138214; HLF_1, human lung fibroblasts; HLF_2, foreskin fibroblasts; NONHSAT138214, the number of lincRNAs data set reported by Cabili and Trapnell (PubMed PMID: 21890647)
