# Supplementary material for: A New Role for LOC101928437 in Non-Syndromic Intellectual Disability: Findings from a Family-Based Association Test
Source: PLoS One. 2015 Aug 19;10(8):e0135669. doi: 10.1371/journal.pone.0135669 (PMC4545728; doi:10.1371/journal.pone.0135669)
Supplement: S5 Table — (DOCX) [file pone.0135669.s007.docx]

**S5 Table. In silico analysis results for SNPs with significant association in single marker analysis.**

| **SNPs** | **Allele** | **Silicon estimation ^a^** | | | | |
| --- | --- | --- | --- | --- | --- | --- |
|  |  | **TFB site** | **Splice site** | **ORF-finder** | **Estimated function ^b^** | **Conservative domain, similar protein and related function estimation** |
| rs6624142 | C | - | - | 65aa | Nonsynonymous  Val>Ile | SRP1 |
|  | T | - | - | 65aa |  | No changed |
| rs6622044 | A | - | - | 38aa | Nonsynonymous  Arg>Ser | Cytoplasmic domain, Non-Cytoplasmic domain  TMhelix, Transmembrane region |
|  | C | - | Gain an acceptor site | 38aa |  | TMhelix domain loss |
| rs4829463 | G | gain SRY site | - | - | -  - | - |
|  | A | gain C/EBRPb site | - | - |  | - |
| rs3116911 | A | - | - | 40aa | Nonsynonymous  Ile>Val | Cytoplasmic domain, Non-Cytoplasmic domain  TMhelix, Transmembrane region |
|  | G | - | - | 40aa |  | No change |
| rs5929554 | A | - | Changed significant | 64aa |  | Transposase_22, L1 transposable element |
|  | T | - |  | - |  | No change |
| rs12164331 | C | - | - | - |  | - |
|  | T | - | - | - |  | - |
| rs5974392 | T | - | - | 37aa | Nonsense SNP | Non_Cytoplasmic domain, Signal peptide domain with C, H and N regions, signalP-noTM |
|  | G | - | Gain an acceptor site | - |  | - |

Abbreviations: TFB site, transcription factor-binding site analysis; ORF-finder, open reading frame finder; SRP1, Improtin subunit alpha-2-like protein domain, which related to interacelluar trafficking and secretion; SRY site, sex-determining region Y gene product; C/EBRPb site, CCAAT/enhancer binding protein beta, a nuclear factor for IL-6 expression.

^a^ The promoter prediction did not include since no any promoter were founded out;
